# Chewed out: an experimental link between food material properties and repetitive loading of the masticatory apparatus in mammals

**DOI:** 10.7717/peerj.1345

**Published:** 2015-11-03

**Authors:** Matthew J. Ravosa, Jeremiah E. Scott, Kevin R. McAbee, Anna J. Veit, Annika L. Fling

**Affiliations:** 1 Departments of Biological Sciences, Aerospace and Mechanical Engineering, and Anthropology, University of Notre Dame, Notre Dame, IN, United States of America; 2 Department of Anthropology, Southern Illinois University, Carbondale, IL, United States of America; 3 Department of Biological Sciences, University of Notre Dame, Notre Dame, IN, United States of America

**Keywords:** Mammals, Masticatory system, Food mechanical properties, Diet, Cyclical/repetitive loading, Jaw loading patterns, Chewing parameters, Mandibular morphology, Rabbits

## Abstract

Using a model organism (rabbits) that resembles a number of mammalian herbivores in key aspects of its chewing behaviors, we examined how variation in dietary mechanical properties affects food breakdown during mastication. Such data have implications for understanding phenotypic variation in the mammalian feeding apparatus, particularly with respect to linking jaw form to diet-induced repetitive loading. Results indicate that chewing frequency (chews/s) is independent of food properties, whereas chewing investment (chews/g) and chewing duration(s), which are proportional to repetitive loading of the jaws, are positively related to food stiffness and toughness. In comparisons of displacement-limited and stress-limited fragmentation indices, which respectively characterize the intraoral breakdown of tough and stiff foods, increases in chewing investment and duration are linked solely to stiffness. This suggests that stiffer foods engender higher peak loads *and* increased cyclical loading. Our findings challenge conventional wisdom by demonstrating that toughness does not, by itself, underlie increases in cyclical loading and loading duration. Instead, tough foods may be associated with such jaw-loading patterns because they must be processed in greater volumes owing to their lower nutritive quality and for longer periods of time to increase oral exposure to salivary chemicals.

## Introduction

Prolonged oral preparation and processing of foodstuffs is a hallmark of mammals. The functional complexity of the jaw-closing muscles and morphological diversity of the dentition are designed to expose the nutritious portion of a food item to salivary chemicals in the oral cavity. Biting and chewing serve to facilitate oral fragmentation, resulting in increasingly smaller food particles that are eventually swallowed and then further exposed to digestive enzymes in the alimentary canal. Apart from dental specializations to augment food fragmentation and maintain tooth function during an organism’s lifetime (Kay, 1975; Lucas, 2004), a mechanically challenging diet may require larger bite and jaw-muscle forces and/or a greater number of chewing cycles to break down a resistant item into sufficiently small pieces for swallowing and further digestion. Variation in jaw-muscle activity patterns affects the magnitude and frequency of muscle, and ultimately bite, forces imparted to a food item and masticatory elements throughout a chewing sequence. For these reasons, it has been long hypothesized that feeding behavior and jaw morphology are dictated by food mechanical properties (Hylander, 1979a; Hylander, 1979b).

Understanding such functional relationships allows us to address evolutionary variation in the mammalian masticatory complex. Indeed, jaw form is frequently employed to make inferences about ecology and feeding behavior in extinct species (e.g., Demes & Creel, 1988; Daegling & Grine, 1991; Janis, 1995; Menegaz et al., 2009; Gill et al., 2014; Scott et al., 2014a; Ravosa et al., 2015). This reflects the large literature linking diet and jaw form in extant species (Hylander, 1979b; Bouvier & Hylander, 1981; Daegling, 1992; Lieberman et al., 2004; Ravosa & Hogue, 2004; Ravosa et al., 2007; Scott, Hogue & Ravosa, 2012; Scott et al., 2014a), and the fact that mandibles are one of the most commonly preserved elements in the fossil record. Nonetheless, the strength of the connection between diet and jaw form has been questioned, highlighting the intervening factors that undermine the generality of such functional associations (Ross, Iriarte-Diaz & Nunn, 2012). Others have underscored the limitations of morphological inference in paleobiology, advocating an experimental and multifactorial perspective for understanding dietary effects on masticatory form (Ravosa et al., 2015).

Thus, despite a sizable body of prior experimental and comparative work, there remain a number of significant gaps in our understanding of the functional links among dietary properties, chewing patterns, and jaw form in living and fossil mammals. Continued difficulty with inferring the adaptive basis of phylogenetic transformations in the feeding apparatus is related to two shortcomings of the current evidence regarding dietary determinants of masticatory form and function. The first is the fact that infrequent, high-magnitude forces imparted to bone are expected to engender the same physiological and evolutionary responses as lower-magnitude, cyclical loading (Bouvier & Hylander, 1981; Lanyon & Rubin, 1985; Biewener, 1993; Turner, Forwood & Otter, 1994). A second and related issue pertains to an incomplete understanding of the impact of food mechanical properties on variation in chewing behaviors that may underlie load-related variation in jaw morphology. Uncertainty in how dietary mechanical properties differentially influence peak loads versus cyclical loading represents a significant impediment to integrating existing experimental, comparative, and ecological information regarding feeding behavior in mammals.

In terms of the respective roles of loading magnitude and repetitive loading on bone formation in the masticatory complex, considerably more experimental information is available regarding the former. In mammals, cortical bone modeling and remodeling in the mandible is associated with routine oral processing of stiff and/or tough foods (e.g., Beecher & Corruccini, 1981; Bouvier & Hylander, 1981; Bouvier & Hylander, 1996; He & Kiliaridis, 2003; Lieberman et al., 2004; Ravosa et al., 2007; Ravosa et al., 2015; Scott et al., 2014a; Scott et al., 2014b). This research shows that postnatal variation in diet-related jaw-loading patterns has a marked influence on masticatory bone formation on par with levels of morphological variation between sister taxa that vary in dietary proclivities in the wild (Scott et al., 2014a; Scott et al., 2014b; Ravosa et al., 2015).

In those mammals for which there are sufficient intraspecific data to evaluate the influence of food mechanical properties on variation in peak-strain magnitudes along the mandible, higher bone-strain levels tend to occur during the postcanine chewing and biting of tough and/or stiff foods in comparison to weak, brittle foods (Table 1). Bone strain on the working (i.e., chewing) side of the mandible is proportional to variation in bite-force magnitudes (Weijs & De Jongh, 1977; Hylander, 1979a; Hylander, 1979b; Hylander, 1986; Hylander et al., 1998; Ross et al., 2007; Ravosa et al., 2010; Rafferty et al., 2012). This finding, coupled with field data regarding food properties and bite forces in bamboo lemurs (Vinyard, Yamashita & Tan, 2008) and peccaries (Kiltie, 1982), highlights the positive association between dietary properties and peak masticatory loads. Finally, experimental and comparative data indicate that mammals that routinely ingest stress-limited (i.e., stiff) and/or displacement-limited (i.e., tough) foods typically exhibit relatively larger jaws to counter elevated peak masticatory stresses (e.g., Freeman, 1979; Hylander, 1979b; Bouvier, 1986; Biknevicius & Ruff, 1992; Daegling, 1992; Spencer, 1995; Ravosa & Hogue, 2004; Hogue, 2008; Taylor, Vogel & Dominy, 2008; Vinyard, Yamashita & Tan, 2008). A similar argument exists regarding variation in mandibular symphyseal fusion in mammals (Scott, Hogue & Ravosa, 2012).

**Table 1:** Mean values for mandibular peak-strain data in mammals (available only for rabbits and primates) recorded while processing various experimental foods, with corresponding mean food mechanical properties and fragmentation indices^a^.

Species	Food	W–S corpus shear strain^b^ (*γ*)	Elastic modulus^c^ (*E*, MPa)	Toughness^d^ (*R*, Jm^−2^)	Stress-limited index^e^ ([*E*∗*R*]^0.5^)	Displacement- limited index^e^ ([*R*/*E*]^0.5^)
Rabbit	Hay (wet/dry)	604	227.8/3,335.6	1,759.2/2,759.8	633.0/3,034.1	2.8/0.9
(*Oryctolagus*)	Pellet	590	29.2	1,030.6	173.5	5.9
	Carrot	297	6.9	343.9	48.7	7.1
Galago	Monkey chow	1,459	50.4	1,030.6	228.0	4.5
(*Otolemur*)	Gummy bear	1,140	0.1	1,709.7	10.9	156.3
	Prune	565	0.5	345.7	12.8	27.1
	Raisin	494	0.2	306.6	8.2	37.3
Owl monkey	Gummy bear	1,107	0.1	1,709.7	10.9	156.3
(*Aotus*)	Prune	1,063	0.5	345.7	12.8	27.1
	Apple skin	733	12.9	662.9	92.4	7.2
Macaque	Monkey chow	775	50.4	1,030.6	228.0	4.5
(*Macaca*)	Popcorn kernel	705	325.4	2,978.8	984.5	3.0
	Apple skin	509	12.9	662.89	92.4	7.2

**Notes.**

a By definition, stiff items exhibit a high elastic modulus (*E*). Because such food items experience little strain at high stresses, they influence oral fragmentation in a particular way characterized as stress-limited (Lucas, 2004; Williams et al., 2005). In contrast, when a food item exhibits greater toughness (*R*) and thus requires higher strains to fragment, oral breakdown of the bolus is displacement-limited. Fragmentation indices for displacement-limited ([*R*/*E*]^0.5^) and stress-limited ([*E*∗*R*]^0.5^) foods reflect the toughness and stiffness, respectively, of an item.

b Rabbit data for the working-side (W-S) mandibular corpus are from Weijs & De Jongh (1977), while similar data for primates are from Hylander et al. (1998).

c Data for hay and pellets are from Ravosa et al. (2007), while the remaining data are from Williams et al. (2005).

d Data for hay are from Ravosa et al. (2007), whereas the remaining data are from Williams et al. (2005). Data for monkey chow are used to represent toughness for pellets.

e Data are calculated based on mean values for stiffness and toughness presented in this table.

**Figure 1:**
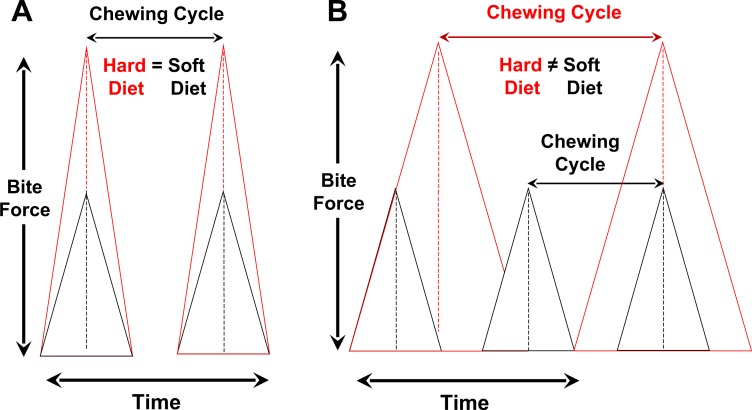
Relationships among bite-force magnitude, chewing cycle length and chewing frequency when the latter two parameters are the same for hard or tough vs. soft foods (A) or different between such foods (B). (A) Rate of force generation (= slope) is modified to produce a larger peak bite force for hard foods, with chewing cycle and chewing frequency being similar. In this scenario, chewing frequency will vary independent of chewing duration (and also chewing investment). (B) Rate of force generation is constant, but chewing cycle and chewing frequency differ due to the disparity in peak forces used to process hard/tough vs. soft foods. In this case, as chewing frequency is lower during the processing of hard/tough foods, this will also result in consequent increases in overall chewing duration. *In vivo* strain data for mammals support the model at left where chewing frequency is independent of food properties and peak bite-force magnitudes (Ross et al., 2007).

**Figure 2:**
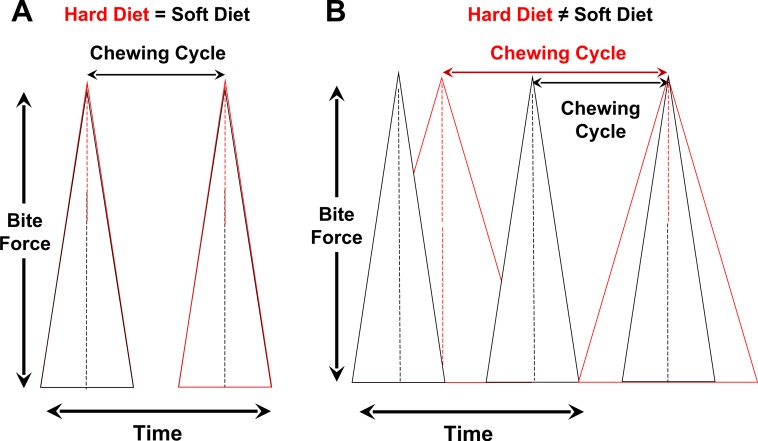
Controlling for variation in bite-force magnitudes, hypothesized relationships between chewing cycle length and chewing frequency when both parameters are the same for hard/tough vs. soft foods (A) or different between such foods (B). (A) Chewing cycle and chewing frequency are both similar, which means that increases in chewing duration of hard/tough foods are due solely to greater chewing investment and, in turn, cyclical loading. (B) Rate of force generation to orally process hard/tough vs. soft foods differs, such that chewing cycle and chewing frequency differ. In this scenario, chewing duration will be positively correlated with chewing cycle length and inversely related to chewing frequency.

During mastication of mechanically challenging food items, the generation of a larger bite force during a chewing cycle can be attained by increasing the rate of force generation (Fig. 1A) and/or by increasing the time to peak force (Fig. 1B). In the former case, chewing frequency is independent of dietary properties, due perhaps to constraints on the neuromotor control of rhythmic jaw movements (e.g., Ross et al., 2007). In the latter scenario, chewing frequency is inversely correlated with food properties, which results in greater overall chewing duration incurred processing tough and stiff items. Food mechanical properties can also positively affect chewing duration by modulating chewing investment, which is the number of chewing cycles during oral breakdown of a given amount of food (Figs. 1 and 2A). Variation in all three chewing parameters (*frequency, investment, duration*) directly determines the loading environment experienced by masticatory elements like the mandible. Diet-induced alterations in jaw-loading patterns are known to influence bone formation throughout the oral cavity, which maps onto evolutionary variation in masticatory structures (Bouvier & Hylander, 1981; Lieberman et al., 2004; Ravosa et al., 2007; Ravosa et al., 2015; Menegaz et al., 2009; Scott et al., 2014a; Scott et al., 2014b).

Independent of variation in bite-force levels, there are two ways that dietary properties are hypothesized to affect cyclical or repetitive loading (Fig. 2). One is by modulating *chewing frequency*, which would alter the number of chewing cycles over a given time period and thus potentially increase the length of a chewing sequence or *chewing duration*. The second way to influence cyclical loading is via the alteration of *chewing investment*, which would modify the number of chewing cycles per unit of food mass (i.e., chews/gram) and thus affect chewing duration; this need not result in the alteration of chewing frequency.

Cyclical loading is commonly invoked to explain mandibular robusticity in mammals (Hylander, 1979b; Hylander, 1988; Bouvier & Hylander, 1981; Demes & Creel, 1988; Daegling & Grine, 1991; Ravosa & Hogue, 2004; Laden & Wrangham, 2005; Hogue, 2008; Janis et al., 2010; Scott et al., 2014a; Scott et al., 2014b). However, in contrast to data on the role of peak loads, there is no empirical evidence regarding the extent to which toughness and stiffness influence variation in the number of chewing cycles required to orally process a given food item and, thus, how these mechanical properties affect the duration of a given chewing sequence (Fig. 2). This surprising lack of key behavioral data on cyclical loading for any mammal represents a critical gap in our knowledge of feeding biology that has hindered interpretations of phenotypic variation in diverse living and extinct taxa. For example, data regarding the role of dietary properties on cyclical loading are central for assessing ongoing debate about hominin evolution (Daegling et al., 2011; Daegling et al., 2013; Strait et al., 2013; Macho, 2014; Scott et al., 2014a; Smith et al., 2015) and differences in jaw form between artiodactyls and perissodactyls (Janis et al., 2010). In this study, we examine how variation in dietary mechanical properties affects cyclical loading during mastication in the New Zealand white rabbit (*Oryctolagus cuniculus*). Our results provide the first experimental evidence linking cyclical loading, long-argued to be a major determinant of jaw form in living and fossil mammals, to a specific food mechanical property.

## Materials & Methods

### Sample

Our sample consisted of adult white rabbits. This species is an excellent model organism because it resembles many other mammalian herbivores in functionally important aspects of its physiology and chewing behavior, including cortical bone modeling and remodeling, transverse jaw movements, elevated levels of balancing-side jaw-adductor muscle force, and diet-related covariation in jaw-adductor muscle activity and mandibular bone-strain patterns (e.g., Herring & Scapino, 1973; Weijs & De Jongh, 1977; Weijs & Dantuma, 1981; Hylander, Johnson & Crompton, 1987; Hylander, Johnson & Crompton, 1992; Hylander et al., 1998; Hylander et al., 2000; Weijs, Brugman & Grimbergen, 1989; Hirano et al., 2000; Herring et al., 2001; Herring et al., 2008; Rafferty et al., 2012). The masticatory apparatus of *O. cuniculus* also conforms to the morphological pattern seen in many other herbivores in possessing a jaw joint elevated high above the occlusal plane that is capable of rotational and translational movements, a vertically deep face, limited gape, and an anteriorly positioned masseter muscle.

Adulthood was defined as skeletal maturity, which is attained at about six months of age in this species (Sorensen, Rogers & Baskett, 1968; Yardin, 1974). All subjects were housed at the University of Notre Dame’s (ND) animal care facility, Freimann Life Science Center, which is USDA-licensed, AAALAC-accredited, and subject to periodic inspections. All procedures used in this study were approved by ND’s Institutional Animal Care and Use Committee (protocol# 14-04-1739). Day-to-day care of the animals, including monitoring of their health, was handled by trained veterinary staff.

### Data

The experimental foods used in this study included rabbit pellets, carrots, and hay. The effects of these foods on variation in mandibular strain patterns recorded *in vivo* for rabbits are well known (Table 1). In comparison to carrots, hay and pellets result in greater jaw-muscle activity and higher mandibular bone strain in rabbits (Weijs & De Jongh, 1977; Weijs & Dantuma, 1981; Weijs, Brugman & Grimbergen, 1989), with consequent diet-induced increases in the proportions of masticatory structures during postweaning ontogeny (Ravosa et al., 2007; Menegaz et al., 2009; Scott et al., 2014a; Scott et al., 2014b). The mechanical properties of these foods fall within the range of values for foods ingested by, for example, wild primates (Williams et al., 2005), which also exhibit diet-related covariation in jaw-adductor muscle activity and mandibular bone-strain patterns (Table 1; Hylander, Johnson & Crompton, 1987; Hylander, Johnson & Crompton, 1992; Hylander et al., 1998; Hylander et al., 2000).

Prior data on the mechanical properties of the experimental foods indicate that hay is the most mechanically challenging of the three, being tougher (higher fracture toughness, *R*) and stiffer (higher elastic modulus, *E*) than pellets and carrots, with pellets being tougher and stiffer than carrots (Williams et al., 2005; Ravosa et al., 2007) (Table 1). Wet hay, which models exposure to saliva, is an order of magnitude stiffer than pellets, while dry hay has an elastic modulus two orders of magnitude greater than pellets (Ravosa et al., 2007). Carrots are significantly less tough and less stiff than pellets and especially hay (Table 1). The toughness of small rabbit pellets is technically difficult to quantify, but a reasonable estimate is the toughness value for monkey chow, which has an elastic modulus roughly similar to that of pellets (Table 1) (Williams et al., 2005; Ravosa et al., 2007). Using stiffness and toughness values, we calculated stress-limited ([*R*∗*E*]^0.5^) and displacement-limited ([*R*/*E*]^0.5^) fragmentation indices, which reflect the mechanical conditions dictating the rate of intraoral breakdown of a given food item due respectively to its stiffness and toughness (Lucas, 2004; Williams et al., 2005).

Feeding experiments typically occurred in the morning as part of the daily feeding schedule and did not require prior fasting. As data collection opportunistically employed rabbits from other ongoing projects, not all rabbits received the three food types, but each subject received at least two. Therefore, sample sizes vary for each food. Behavioral observations consisted of a feeding bout where a single rabbit was initially offered a given mass (g) of a particular food. The duration of a bout was considered to be paused when the subject stopped chewing and restarted if the subject began chewing again. The chewing bout was considered finished when the subject stopped chewing for several seconds, swallowed its food, and no longer expressed interest in any remaining food, which was collected and weighed to determine the amount consumed. The length of time spent chewing is referred to as *chewing duration* (s). Once the initial feeding bout ended, the subject was then fed a quantity of a second experimental food that was equal in mass to the amount of the first experimental food that the subject consumed; this procedure was then repeated for the third experimental food (for subjects that received all three), although in some cases feeding experiments were completed on consecutive days. The bolus size of all three foods was roughly similar. Thus, while the quantity of food ingested varied among rabbits, it did not vary for a given subject over the course of a given suite of experiments.

In all cases, a subject was filmed at 60 frames per second using a digital video recorder. The video footage was used to obtain an additional chewing parameter during the processing segment of each feeding bout: *chewing frequency* (chews/s). Feeding sequences represent earlier phases of mastication—i.e., once a food bolus was formed intraorally and jaw closing was observed—and consisted of a minimum of 10 chewing cycles. Chewing frequency was averaged over a given feeding bout, as defined above. A third parameter was computed by multiplying chewing frequency by chewing duration for each feeding bout and dividing the product by the consumed food mass. This parameter is therefore the number of chews scaled by the amount of food consumed and is referred to as *chewing investment* (chews/g). The raw data collected from these experiments are available in Tables S1–S3.

### Analysis

We initially examined our data by comparing chewing parameters within individuals using ratios that expressed a given parameter for one type of food versus the same parameter for another food. Such ratios were computed with the more mechanically challenging food as the numerator: e.g., hay chewing investment divided by pellet chewing investment. Because only some individuals received all three food types, the number of individuals for which each ratio could be calculated varied: hay-pellet ratios, *n* = 14; pellet-carrot ratios, *n* = 13; hay-carrot ratios, *n* = 4. These within-subject ratios facilitated a critically important control of between-individual variation in functional ability to chew foods of different mechanical properties, absent from prior experimental studies of chewing patterns (e.g., Hylander, Johnson & Crompton, 1992; Hylander et al., 1998; Hylander et al., 2000; Ross et al., 2007). Such information reveals functional patterns that are potentially obscured when incomplete behavioral and physiological data are pooled for intraspecific and interspecific analyses of dietary effects.

Within each of the three chewing parameters, these ratios are directly comparable among individual subjects and were pooled for analysis. To evaluate whether a given ratio differed significantly from 1.0, we generated 95% confidence limits for the mean ratio using the bootstrap (percentile method) with 1,000 iterations. A ratio not significantly different from 1.0 indicates the absence of a dietary influence on that chewing variable. Due to small sample size (*n* = 4), confidence limits were not used for hay-carrot comparisons; in this instance, the range of values is presented. In cases where a ratio differed from 1.0, we evaluated the relationship of the corresponding parameter with the food mechanical properties (*E*, *R*) and fragmentation indices ([*E*∗*R*]^0.5^, [*R*/*E*]^0.5^; see Table 1) using a linear mixed-effects model, with subject entered as a random effect. This procedure was used because it can handle samples consisting of repeated observations of the same individuals along with missing data (Baayen, Davidson & Bates, 2008), both of which present problems for standard ANOVA and regression approaches. Linear mixed-effects models were computed using the nlme package (version 3.1–120; Pinheiro et al., 2015) for R (R Core Team, 2014). Data were logged (base *e*) for analysis to reduce heteroscedasticity and to improve the linear relationship between mechanical properties and chewing parameters.

## Results

Our data indicate that chewing frequency does not vary among the experimental foods. As shown in Table 2, within-individual ratios of chews per second for one food versus another are not statistically different from 1.0. On the other hand, our findings show that chewing investment and chewing duration differ among the experimental foods. Within-individual ratios of chewing investment and chewing duration for one food versus another are statistically different from 1.0 in all cases, with more mechanically challenging items requiring more time and investment in each comparison (Table 2). These disparities in time and investment range from pellets having values that are approximately twice as large as those for carrots, to hay having values that are 8–9 times greater than those for carrots. Note that because chewing frequency is similar among the foods, chewing duration and chewing investment are redundant measures, given that the latter parameter is computed using the former along with chewing frequency (and scaled by food mass). Accordingly, we focus on chewing investment in the remainder of the analysis.

**Table 2:** Means, sample sizes, and 95% confidence intervals for within-individual chewing-parameter ratios^a^.

Comparison	Chewing frequency (chews/s)	Chewing duration (s)	Chewing investment (chews/g)
Hay/carrots	0.96 (*n* = 4, 0.84–1.11)	9.02 (*n* = 4, 7.10–10.81)	8.53 (*n* = 4, 7.47–9.67)
Hay/pellets	0.99 (*n* = 12, 0.97–1.01)	3.22 (*n* = 14, 2.79–3.68)	2.95 (*n* = 12, 2.58–3.35)
Pellets/carrots	1.06 (*n* = 13, 1.00–1.10)	2.20 (*n* = 13, 1.93–2.52)	2.32 (*n* = 13, 2.01–2.61)

**Notes.**

a Due to small sample sizes, confidence limits were not generated for hay-carrot comparisons; in this instance, the range of values is presented.

**Figure 3:**
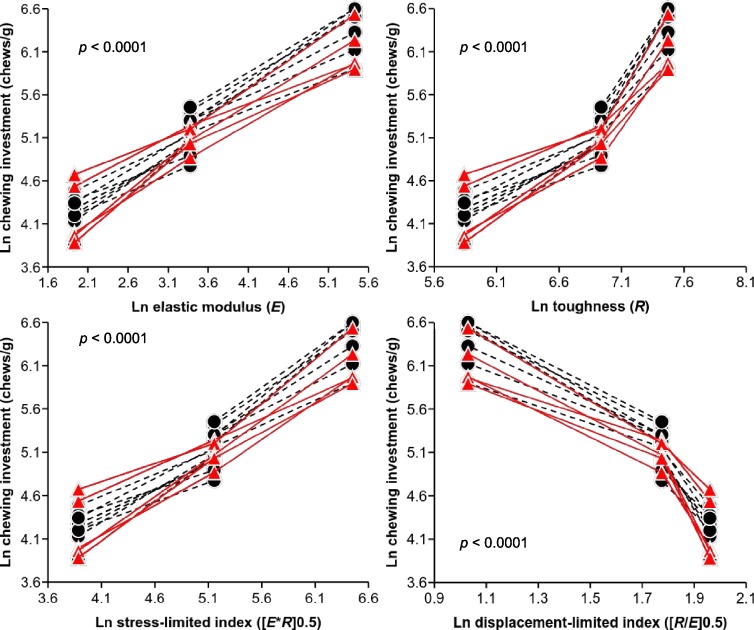
Relationships between chewing investment (chews/g) and food mechanical properties (elastic modulus, *E*; toughness, *R*) and oral fragmentation indices (stress-limited, [*E*∗*R*]^0.5^; displacement-limited, [*R*/*E*]^0.5^). *P*-values generated using a linear mixed-effects model are provided. All data are logged (base *e*). Black circles represent subjects that were given only two of the experimental foods; the values for each of these subjects are connected with dashed black lines. Red triangles indicate individuals that were given all three experimental foods; the values for each of these subjects are connected with solid red lines. Note that individuals with three data points are similar to individuals represented by only two data points.

Figure 3 illustrates the relationships between individual chewing investments and the mean food mechanical properties and fragmentation indices. Here, we are comparing chewing investment rather than ratios of chewing investments for particular foods. Because chewing investment is scaled by food mass, it can be compared across individuals, unlike chewing duration. Analysis of these data using the linear mixed-effects model indicates that chewing investment is positively and significantly (*p* < 0.0001) related to elastic modulus (*E*), toughness (*R*), and the stress-limited index ([*R*∗*E*]^0.5^), but inversely related (*p* < 0.0001) to the displacement-limited index ([*R*/*E*]^0.5^). All individuals exhibit this pattern, but note that there is between-individual variation in chewing investment within each food type. It is also apparent that the relationships involving *R* and the displacement-limited index are not strictly linear, but the directions of their associations with chewing investment are nonetheless clear.

## Discussion

There are two ways that dietary properties might influence repetitive loading of the jaws. One is by modulating chewing frequency, which would alter the number of chewing cycles over a given time period and thus potentially affect the duration of a chewing sequence (Figs. 1 and 2). The second means of influencing repetitive loading is via the alteration of chewing investment, which would modify the number of chewing cycles per unit of food mass (i.e., chews per gram) and ultimately influence overall time spent chewing (i.e., chewing duration); this need not result in the alteration of chewing frequency. Of course, some combination of both effects is possible.

Our analyses indicate that chewing frequency did not vary among the experimental foods (Table 2), averaging about 4 Hz for each of three mechanically distinct foods. These values are comparable to those observed in a prior investigation of rabbit chewing behavior (Anderson, Hector & Linden, 1985). Because previous studies have not employed strict control of behavioral and dietary data for subjects used to characterize a given species, our findings provide perhaps the best support to date for the hypothesis that chewing frequency is independent of variation in food properties (Ross et al., 2009a; Ross et al., 2009b). This observation likewise strongly supports an earlier suggestion that the generation of greater occlusal forces, which vary in proportion to chewing cycle and jaw loading/unloading, occurs largely by increasing the rate of force production, rather than by lengthening the duration of a given chewing cycle and thus decreasing chewing frequency (Ross et al., 2007) (Fig. 1).

In contrast to the results for chewing frequency, there is a clear relationship between food mechanical properties and chewing investment and duration (Table 2, Fig. 3). First, hay, which is the stiffest and toughest food examined herein, required more chews per gram and therefore relatively longer chewing bouts than pellets and carrots. Second, pellets, which are stiffer and tougher than carrots, required more chews per gram and longer chewing bouts than carrots. Unexpectedly, comparisons of oral fragmentation indices for the experimental foods indicate that while higher stress-limited indices uniformly characterize foods that require more chews per gram, displacement-limited indices are, in contrast to predictions, *lower* for such foods. In other words, whereas chewing investment increases with stiffness, toughness, and the stress-limited index, the displacement-limited index is negatively related to chewing investment (Fig. 3). This contrast between the displacement-limited index and the other variables suggests that chewing investment—and by extension, chewing duration—is driven primarily by the elastic modulus.

However, chewing investment is also positively associated with *R*, and the stress-limited index is computed using both *E* and *R*. These two variables are also correlated among the foods used in this study (i.e., as *E* increases from carrot to hay, so does *R*; Table 1). Therefore, to further evaluate the influence of food mechanical properties on chewing investment, we constructed a linear mixed-effects model with *E* and *R* both entered into the analysis as independent variables. The results of this test support the conclusion that elastic modulus is the primary influence on variation in chewing investment in our sample: *E* remains significantly positively related to chewing investment (*p* < 0.0001) in this model, whereas *R* is no longer significant (*p* = 0.4259). This result does not depend on the order in which *E* and *R* are entered into the model. Taken as whole, our results reveal the singular importance of stiffness (elastic modulus) as a determinant of diet-related disparities in chewing patterns for rabbits.

### Reconstructing feeding behavior in extinct species

As noted in the introduction, one of the motivations for our experimental work on rabbit feeding biology is to refine and improve the neontological comparative framework that is used to link phenotypic variation in jaw morphology to ecology and behavior in fossil species (Ravosa et al., 2007; Ravosa et al., 2015; Menegaz et al., 2009; Scott et al., 2014a; Scott et al., 2014b). Based on the well-established positive association between bite-force magnitudes and food stiffness in extant mammals (Weijs & De Jongh, 1977; Hylander, 1979a; Hylander, 1979b; Hylander, 1986; Hylander et al., 1998; Ross et al., 2007; Ravosa et al., 2010; Rafferty et al., 2012), increases in jaw robusticity can be reasonably interpreted as an evolutionary or plastic response to generating high-magnitude loads in cases where other lines of evidence suggest that hard objects (e.g., seeds, nuts) are a component of a fossil species’ diet. However, given that our data implicate the elastic modulus, and thus the stress-limited characteristics of hard, stiff foods, as an important determinant of cyclical loading of the jaws in mammals, it is likely that heavily buttressed jaws of suspected hard-object-feeding fossil species also reflect repetitive loading. In other words, attempts to tie the jaw morphology of fossil hard-object feeders to either high-magnitude forces or repetitive loading may not be possible, because both of these regimes play a role in breaking down such foods. Moreover, while it is often assumed that repetitive loading is linked primarily with dietary toughness (e.g., Hylander, 1979b; Hylander, 1988; Demes & Creel, 1988; Daegling & Grine, 1991; Lucas, 2004; Daegling et al., 2011; Ungar & Sponheimer, 2011), our data challenge this assumption.

Although our results indicate that increasing toughness, by itself, does not appear to be associated with greater chewing investment, we are hesitant to reject a link between tough foods and repetitive loading and concomitant increases in jaw robusticity in mammals. Rather, we hypothesize that nonmechanical factors may lead to greater cyclical loading of the jaws of species with diets that can be generally characterized as tough, such as grazers, browsers, and folivores. Many tough foods are recognized as being relatively low in quality, with reduced nutritive properties and potential limits on digestibility, requiring consumption in large quantities to meet basic metabolic and nutritional demands. Shifting to such a diet from one that is more nutrient-rich will result in increased intraoral processing and greater repetitive loading of masticatory elements, resulting in a more robust mandible (see also Macho, 2014). According to this line of reasoning, there is an indirect relationship between dietary toughness and repetitive loading that is mediated by a correlation between toughness and nutritional properties in low-quality foods. This suggestion is consistent with behavioral evidence that herbivores that rely on tough, low-quality foods spend a relatively large portion of each day engaged in feeding activities (Demment & Van Soest, 1985; Sailer et al., 1985; Wright et al., 2008). Thus, displacement-limited and stress-limited foods may both lead to similar levels of repetitive loading and increases in jaw robusticity but for different reasons. This observation presents a challenge with regard to using jaw form to make specific inferences about feeding behavior in fossil species (Ross, Iriarte-Diaz & Nunn, 2012), and it highlights the need to consider multiple lines of evidence (Ravosa et al., 2015).

### Dental morphology and cyclical loading

One issue not addressed by our study is the potentially important role that dental form plays in chewing investment and cyclical loading. Mammals that masticate tough leaves tend to have long, well-developed molar shearing crests, whereas species that exploit hard fruits and seeds tend to have bunodont teeth with much shorter and blunter crests (Kay, 1975; Kay, 1978; Walker & Murray, 1975; Sheine & Kay, 1977; Sheine & Kay, 1982; Strait, 1998; Hogue & ZiaShakeri, 2010). Because “the working surface of the molars have a strong effect on the physiological rate of breakdown” (Lucas, 2004, p. 167), it is likely that variation in molar relief translates into intra- and interspecific disparities in chewing investment for different types of foods. Specifically, we speculate that taxa with trenchant shearing crests will be more effective at processing displacement-limited foods (i.e., chewing investment will be lower) than they are at processing stress-limited foods. For such species, the stress-limited index of a given food may be a greater determinant of chewing investment. Our results are consistent with this idea: rabbits have well-developed postcanine shearing blades (e.g., Von Koenigswald et al., 2010) like other mammalian herbivores, and they are, according to our data, limited more by the elastic modulus than by dietary toughness. A priority for future research should be to collect similar data on chewing behavior in a mammal with bunodont molars to evaluate whether the converse pattern holds—that such species are more effective at processing stress-limited foods versus displacement-limited foods, and that dietary toughness differentially drives chewing investment. Interestingly, it has been argued that prolonged milling and grinding in robust australopiths would have been necessary when consuming tough foods due to the fact that their low-cusped molars are poorly designed for shearing such items vis-a-vis the high-cusped molars of extant primate folivores (Ungar et al., 2010).

Thus, apart from being directly linked to food mechanical properties, cyclical loading due to higher chewing investment might be a consequence of a molar shape that is less effective at fracturing certain mechanically challenging foods (due to functional trade-offs or phylogenetic constraints). We were not able to incorporate data on occlusal relief and crest development for the experimental subjects included this study, but future investigations might use such information to inform inter- and intraspecific analyses of diet, food breakdown, and chewing patterns. By exploring direct and indirect effects of food properties on cyclical loading, this integrative perspective will increase our understanding of the complex links among diet, feeding performance, and mammalian skull form. Development of such a multivariate approach is requisite for unraveling the hierarchical suite of effects on masticatory form and function, an ongoing challenge that continues to hinder advances in functional and evolutionary research on chewing patterns such as cyclical loading (Ravosa et al., 2015).

## Conclusions

Analysis of chewing patterns in rabbits suggests that repetitive loading in mammals is proportional to dietary stiffness, but not related directly to dietary toughness. This conclusion suggests that, while stiffer foods should result in higher peaks loads *and* elevated cyclical loading during oral processing, a singularly tough item might only engender loads of greater magnitude. These findings challenge conventional wisdom by demonstrating that toughness does not, by itself, underlie increases in cyclical loading and loading duration. Rather, nutritive factors may explain the apparent link between toughness and repetitive loading. This association may be further modulated by variation in postcanine tooth form. Since intra- and interspecific comparisons have the potential to obscure functional signals clearly evident on an individual scale, which corresponds to the hierarchical level at which selection and functional adaptation occurs, the broader experimental, ecomorphological, and paleobiological implications of diet-related phenotypic patterns require further attention. Additional experimental analyses of variation in the masticatory complex of multiple clades will bring further resolution to our understanding of the relative contribution of loading magnitude and cyclical loading on mammalian skull form.

## Supplemental Information

10.7717/peerj.1345/supp-1 Table S1 Comparison of rabbit chewing patterns for hay versus pellets

10.7717/peerj.1345/supp-2 Table S2 Comparison of rabbit chewing patterns for pellets versus carrots

10.7717/peerj.1345/supp-3 Table S3 Comparison of rabbit chewing patterns for hay versus carrots
