# Supplementary material for: Chewed out: an experimental link between food material properties and repetitive loading of the masticatory apparatus in mammals
Source: PeerJ. 2015 Nov 3;3:e1345. doi: 10.7717/peerj.1345 (PMC4636421; doi:10.7717/peerj.1345)
Supplement: Table S1 [file peerj-03-1345-s001.doc]

Supplemental Table 1. Comparison of rabbit chewing patterns for hay versus pellets

|  | Food  Mass (g) | Chewing Duration (s) | | |  | Chewing Frequency (chews/s) | | |  | Chewing Investment (chews/g) | | |
| --- | --- | --- | --- | --- | --- | --- | --- | --- | --- | --- | --- | --- |
| Subject |  | Hay | Pellets | Hay/Pellets |  | Hay | Pellets | Hay/Pellets |  | Hay | Pellets | Hay/Pellets |
| Ch | 7.29 | 1490 | 377 | 3.95 |  | 3.95 | 3.84 | 1.03 |  | 807.53 | 198.64 | 4.07 |
| Tt | 11.85 | 2175 | 659 | 3.30 |  | 4.11 | 4.20 | 0.98 |  | 754.23 | 233.68 | 3.23 |
| Tz | 4.38 | 924 | 177 | 5.22 |  |  |  |  |  |  |  |  |
| Pt | 0.33 | 150 | 37 | 4.05 |  |  |  |  |  |  |  |  |
| S4 | 1.16 | 190 | 51 | 3.73 |  | 4.27 | 4.20 | 1.02 |  | 699.40 | 184.66 | 3.78 |
| S5 | 1.97 | 268 | 103 | 2.60 |  | 4.11 | 3.88 | 1.06 |  | 559.71 | 202.97 | 2.76 |
| S6 | 3.05 | 288 | 157 | 1.83 |  | 4.10 | 4.08 | 1.01 |  | 387.12 | 209.77 | 1.85 |
| S8 | 2.76 | 436 | 142 | 3.07 |  | 4.22 | 4.37 | 0.97 |  | 666.67 | 224.75 | 2.97 |
| S9 | 3.24 | 361 | 149 | 2.42 |  | 4.09 | 4.37 | 0.94 |  | 455.71 | 200.97 | 2.27 |
| S10 | 3.49 | 311 | 145 | 2.14 |  | 4.10 | 4.20 | 0.98 |  | 365.36 | 174.50 | 2.09 |
| Y1 | 1.82 | 193 | 62 | 3.11 |  | 3.70 | 3.82 | 0.97 |  | 392.36 | 130.13 | 3.02 |
| Y2 | 2.98 | 488 | 131 | 3.73 |  | 4.20 | 4.13 | 1.02 |  | 687.79 | 181.55 | 3.79 |
| Y3  Y4 | 3.20  3.30 | 382  295 | 113  117 | 3.83  2.52 |  | 4.27  4.06 | 4.51  4.33 | 0.95  0.94 |  | 509.33  362.71 | 159.15  153.37 | 3.20  2.37 |
| Mean | 3.41 | 568 | 173 | 3.22 |  | 4.10 | 4.16 | 0.99 |  | 474.85 | 161.01 | 2.95 |
| 95% CI |  |  |  | 2.79–3.68 |  |  |  | 0.97–1.01 |  |  |  | 2.58–3.35 |
