# Supplementary material for: Chewed out: an experimental link between food material properties and repetitive loading of the masticatory apparatus in mammals
Source: PeerJ. 2015 Nov 3;3:e1345. doi: 10.7717/peerj.1345 (PMC4636421; doi:10.7717/peerj.1345)
Supplement: Table S2 [file peerj-03-1345-s002.doc]

Supplemental Table 2. Comparison of rabbit chewing patterns for pellets versus carrots

|  | Food  Mass (g) | Chewing Duration (s) | | |  | Chewing Frequency (chews/s) | | |  | Chewing Investment (chews/g) | | |
| --- | --- | --- | --- | --- | --- | --- | --- | --- | --- | --- | --- | --- |
| Subject |  | Pellets | Carrots | Pellets/Carrots |  | Pellets | Carrots | Pellets/Carrots |  | Pellets | Carrots | Pellets/Carrots |
| R1 | 3.03 | 116 | 48 | 2.42 |  | 3.91 | 4.26 | 0.92 |  | 149.69 | 67.49 | 2.22 |
| R4 | 3.02 | 123 | 74 | 1.66 |  | 4.14 | 3.61 | 1.15 |  | 168.62 | 88.46 | 1.91 |
| R5 | 3.00 | 92 | 58 | 1.59 |  | 3.87 | 3.45 | 1.12 |  | 118.62 | 66.70 | 1.78 |
| R7 | 2.99 | 100 | 65 | 1.54 |  | 3.99 | 3.66 | 1.09 |  | 133.44 | 79.57 | 1.68 |
| R8 | 3.01 | 118 | 59 | 2.00 |  | 4.41 | 3.92 | 1.13 |  | 172.88 | 76.84 | 2.25 |
| R10 | 3.03 | 114 | 36 | 3.17 |  | 4.17 | 4.06 | 1.03 |  | 156.89 | 48.24 | 3.25 |
| R11 | 3.01 | 113 | 56 | 2.02 |  | 3.92 | 3.85 | 1.02 |  | 147.16 | 71.63 | 2.05 |
| R13 | 2.97 | 114 | 54 | 2.11 |  | 4.16 | 3.43 | 1.21 |  | 159.68 | 62.36 | 2.56 |
| Y1 | 1.82 | 62 | 26.5 | 2.32 |  | 3.82 | 3.73 | 1.02 |  | 130.13 | 54.31 | 2.40 |
| Y2 | 2.98 | 131 | 71 | 1.85 |  | 4.13 | 4.49 | 0.92 |  | 181.55 | 106.98 | 2.70 |
| S6  Y3  Y4 | 6.59  3.20  3.30 | 282  113  117 | 140  36  39 | 2.01  3.14  3.00 |  | 4.42  4.51  4.33 | 4.39  4.68  4.11 | 1.01  0.96  1.05 |  | 189.14  159.15  153.37 | 93.26  52.66  48.57 | 2.03  3.02  3.16 |
|  |  |  |  |  |  |  |  |  |  |  |  |  |
| Mean | 3.23 | 123 | 60 | 2.20 |  | 4.16 | 3.95 | 1.06 |  | 155.89 | 70.80 | 2.32 |
| 95% CI |  |  |  | 1.93–2.52 |  |  |  | 1.00–1.10 |  |  |  | 2.01-2.61 |
