# Supplementary material for: Chewed out: an experimental link between food material properties and repetitive loading of the masticatory apparatus in mammals
Source: PeerJ. 2015 Nov 3;3:e1345. doi: 10.7717/peerj.1345 (PMC4636421; doi:10.7717/peerj.1345)
Supplement: Table S3 [file peerj-03-1345-s003.doc]

Supplemental Table 3. Comparison of rabbit chewing patterns for hay versus carrots

|  | Food  Mass (g) | Chewing Duration (s) | | |  | Chewing Frequency (chews/s) | | |  | Chewing Investment (chews/g) | | |
| --- | --- | --- | --- | --- | --- | --- | --- | --- | --- | --- | --- | --- |
| Subject |  | Hay | Carrots | Hay/Carrots |  | Hay | Carrots | Hay/Carrots |  | Hay | Carrots | Hay/Carrots |
| Y1 | 2.34 | 335 | 31 | 10.81 |  | 3.45 | 4.10 | 0.84 |  | 493.91 | 54.32 | 9.09 |
| Y2  Y3  Y4 | 2.05  3.20  3.30 | 341  382  295 | 48  36  39 | 7.10  10.61  7.56 |  | 4.40  4.27  4.06 | 3.96  4.68  4.11 | 1.11  0.91  0.99 |  | 731.90  509.33  362.71 | 92.72  52.66  48.57 | 7.89  9.67  7.47 |
|  |  |  |  |  |  |  |  |  |  |  |  |  |
| Mean  Range | 2.72 | 339 | 39 | 9.02  7.10–10.81 |  | 4.05 | 4.21 | 0.96  0.84–1.11 |  | 524.46 | 62.07 | 8.53  7.47–9.67 |
